# Supplementary material for: Design of an Integrated Microfluidic Paper-Based Chip and Inspection Machine for the Detection of Mercury in Food with Silver Nanoparticles
Source: Biosensors (Basel). 2021 Nov 30;11(12):491. doi: 10.3390/bios11120491 (PMC8699263; doi:10.3390/bios11120491)
Supplement: Supplementary file 1 [file biosensors-11-00491-s001.zip › biosensors-1471864-supplementary.pdf]

Supplementary Materials:

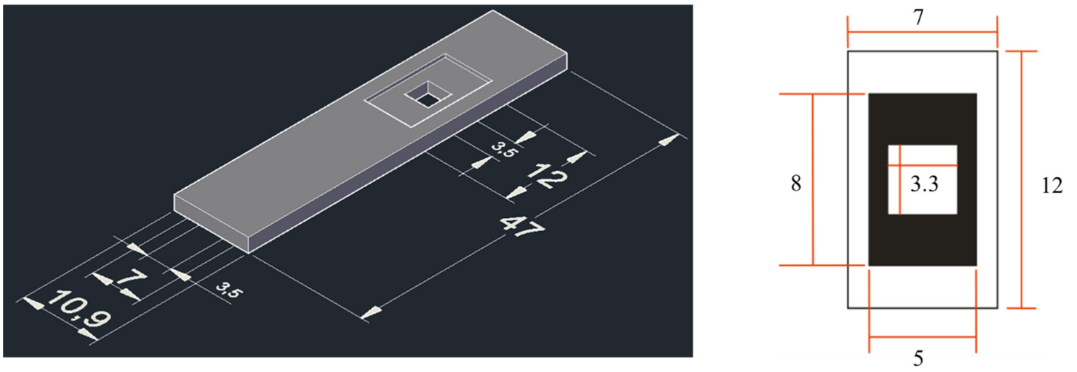

Figure S1. Scheme of paper-based strip (unit: mm).

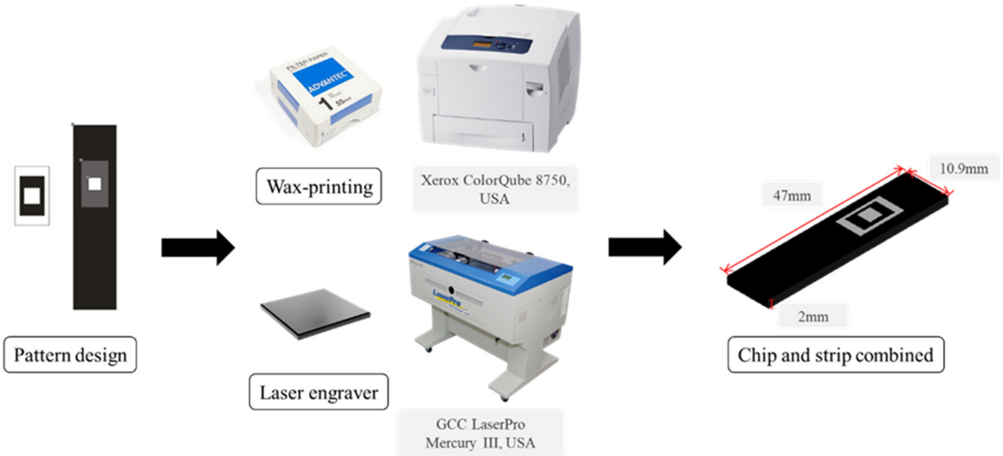

Figure S2. Scheme of manufacturing μPAD paper-based strip and chip. The strip was used as a holder to place the chip in the circle manufactured by a laser engraver. The μPAD paper-based chip was manufactured by a wax printer.

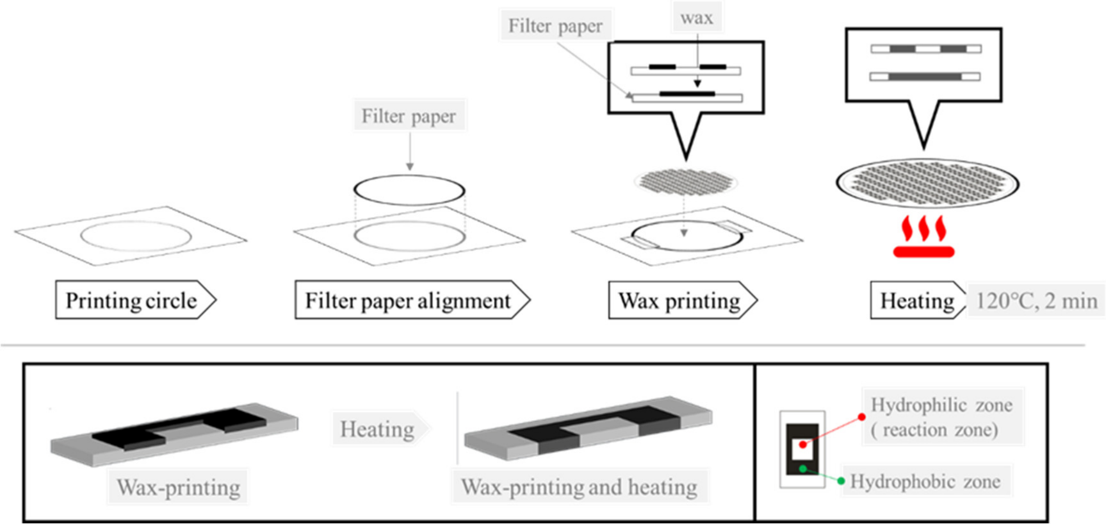

Figure S3. Scheme of manufacturing μPAD paper-based chip. The hydrophobic and hydrophilic zones were manufactured after heating the wax-printed μPAD paper-based chip. The hydrophobic zone could keep the reaction solution in the circle and made the color reaction visible in the circle.

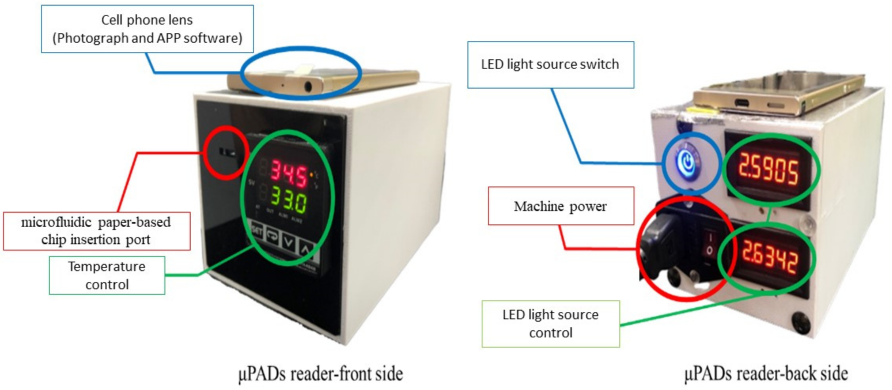

Figure S4. Design and real scheme of μPAD reader.

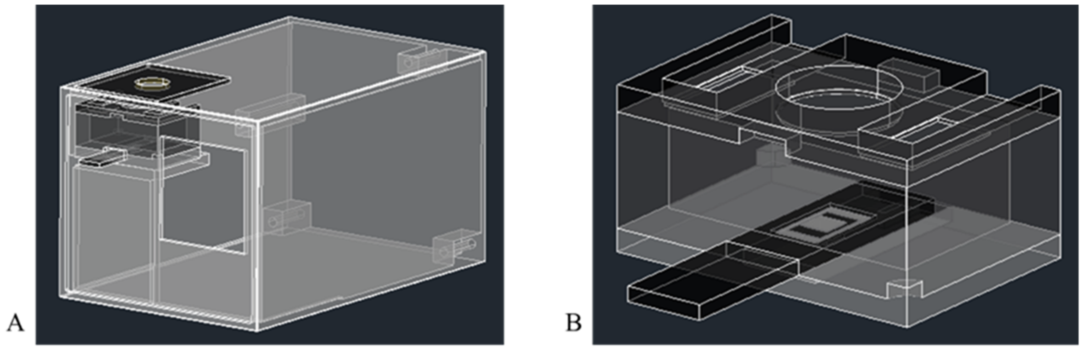

Figure S5. Scheme of μPAD reader box. A is a closed design reader box; B is the photographic space for recording the color change of the paper-based chip inserted into the reader and is also closed to avoid external light.

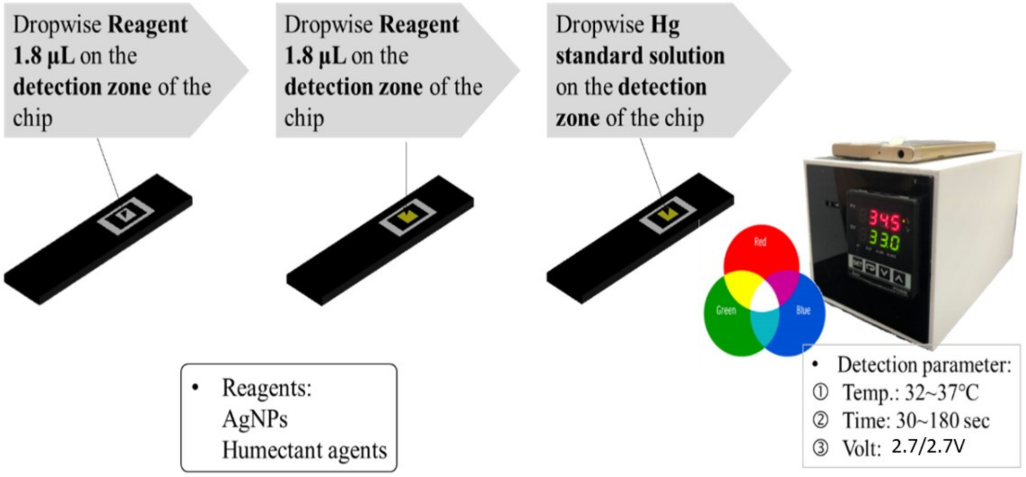

Figure S6. Scheme of μPAD detection procedures.

Table S1. Comparison of detection results for different reaction volume ratios.

Reaction volume ratio (AgNPs: *mercury* standard)	GAP*
1:0.5	23
1:1	20
1:2	12
2:1	41
3:1.5	23

*Reaction volume unit: μL.

*GAP: The resolution gap. The difference between RGB values measured for 0.1 and 2 ppb *mercury* standards.

Table S2. Shelf life test: paper-based chip coated with AgNPs stored for 1–7, 14, 21, and 28 days.

Days	Paper-based chip	GAP*	Regression (R^2)	Days	Paper-based chip	GAP	Regression (R^2)
1.	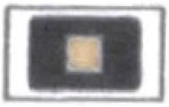	64	0.9996	6	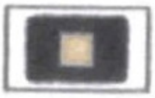	53	0.9955
2	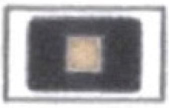	61	0.9992	7	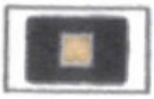	54	0.9949
3	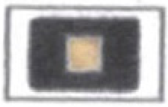	63	0.9991	14	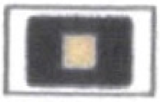	53	0.9916
4	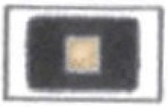	53	0.9987	21	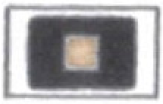	51	0.9867
5	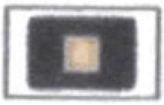	52	0.9976	28	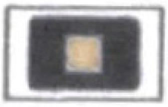	54	0.9792

*GAP: The resolution gap.

Table S3. Method comparison between μPADs, UV–Vis, and CVAAS.

Object \ Methods	μPADs	UV–Vis	CVAAS
Reagents	Silver nitrate<br>Trisodium citrate<br>Sodium borohydride<br>Glycerol<br>Mercury nitrate<br>Nitric acid	Silver nitrate<br>Trisodium citrate<br>Sodium borohydride<br>Mercury nitrate<br>Nitric acid	Sodium borohydride<br>Hydrochloric acid<br>Mercury standard stock<br>Hydrochloric acid
Assay equipment	μPAD reader<br>Filter paper	ELISA reader/UV–Vis spectrophotometer<br>96 wells/cuvette	Cold vapor atomic absorption spectrometer<br>Mercury reaction flask<br>Flowmeter
Time consumed for preparation (HH:MM:SS)	AgNPs prepared 02:00:00<br>Mercury 00:30:00	AgNPs prepared 02:00:00<br>Mercury 00:30:00	Reagent 00:40:00<br>Mercury 01:20:00
Time consumed for standard assay (HH:MM:SS)	02:00:00	01:00:00	02:30:00
Time consumed for sample assay (HH:MM:SS)	00:10:00	00:10:10	00:30:00
Total time consumed (HH:MM:SS)	04:40:00	03:40:00	05:00:00
Limit of detection	0.1 ppb	0.1 ppb	0.25 ppb
Complexity level (1-5)	★☆☆☆☆	★★★☆☆	★★★★☆
Actual efficiency	High	Middle	Low
Cost (NTD)	μPAD chip NTD 1~5	ELISA reader NTD 150,000	CVAAS NTD 300,000~2,000,000

	μPAD reader NTD 3~4000	96 wells NTD 30~60<br>UV–Vis spectrophotometer NTD 100,000~200,000<br>Cuvette NTD 1000~1500	Other equipment NTD 150,000
References	This study	Paper (Vasileva et al., 2017)	Taiwan announcement NIEA W330.52
